# Use of Cefiderocol for Carbapenem-Resistant Gram-Negative Infections in Hospital at Home: Multicentric Real-World Experience

**DOI:** 10.3390/antibiotics14121216

**Published:** 2025-12-03

**Authors:** Andrea Parra-Plaza, Ainoa Ugarte, Eva Benavent, Nicole García-Poutón, Abel Mujal, María Rosa Oltra, Andrés Parra-Rojas, Verónica Rico, Manuel del Río, David Nicolás

**Affiliations:** 1 Hospital at Home Unit, Hospital Clínic Barcelona, Universitat de Barcelona, 08036 Barcelona, Spain; anparra@clinic.cat (A.P.-P.); ugarte@clinic.cat (A.U.); ngpouton@clinic.cat (N.G.-P.); rico@clinic.cat (V.R.); 2 Infectious Diseases Service, Hospital Universitari de Bellvitge, Hospitalet del Llobregat, 08907 Barcelona, Spain; eva.benavent@bellvitgehospital.cat; 3 Hospital at Home Unit, Hospital Universitario Parc Taulí, 08208 Sabadell, Spain; amujal@tauli.cat; 4 Infectious Diseases Unit, Hospital Clínic Universitari de València, 46010 Valencia, Spain; oltra_mar@gva.es; 5 Hospital at Home Unit, Hospital Universitari Vall d’Hebrón, 08035 Barcelona, Spain; andresrodrigo.parra@vallhebron.cat; 6 Hospital at Home Unit, Hospital Universitari Son Espases, 07120 Palma, Spain; manuel.delrio@ssib.es

**Keywords:** Cefiderocol, Gram-negative bacteria, multidrug-resistant infection, hospital at home, outpatient parenteral antibiotic therapy

## Abstract

**Background**: Cefiderocol (CFD) is a novel cephalosporin targeting multidrug-resistant Gram-negative bacterial (GNB) infections. It mimics siderophores to enter into GNB through iron transport receptors. However, evidence on its use in Hospital at Home (HaH) and outpatient parenteral antibiotic therapy (OPAT) programs remains scarce. **Objectives**: The primary objective was to evaluate feasibility and efficacy of CFD in HaH setting. The secondary objective was to assess its safety. **Methods**: A retrospective, observational study was conducted across six Spanish centers between January 2023 and December 2024. Adult patients with documented GNB infections treated with CFD in HaH units were included. Demographic, clinical and microbiological data, treatment characteristics, and outcomes were collected. Statistical analysis was descriptive; no inferential or correlation tests were performed. **Results**: 27 patients were included; 70.4% were male, with a median age of 69 years. Most infections were nosocomial (65.4%), particularly skin and soft tissue (37%). Septic shock occurred in 14.8% of patients. *Pseudomonas aeruginosa* (66.7%) and *Klebsiella pneumoniae* (14.8%) were the most frequent pathogens involved, with Verona Integron-encoded metallo-B-lactamase (VIM, 50%) being the predominant resistance mechanism. CFD was used as a first-line therapy in 63% of cases and in combination with other antibiotics in 40.7%. Median treatment duration was 21.7 days. Administration was mainly via peripherally inserted central catheters (PICC, 33.3%) and electronic pumps (52%). Adverse effects occurred in 7.4% of patients, leading to discontinuation in one case. A total of 88.8% of patients achieved clinical success, with 7.7% recurrence within a month. Escalation of care occurred in 7.7% and 19.2% were readmitted within a month after HaH discharge. No infection-related deaths were reported. **Conclusions**: CFD is a feasible, safe, and effective treatment for difficult-to-treat GNB infections in HaH settings.

## 1. Introduction

Carbapenem-resistant Gram-negative bacterial (GNB) infections have become a severe emergent health threat, particularly for nosocomial settings. These infections are related to increasing morbidity and mortality rates as a consequence of delayed initiation of targeted antibiotic therapy or inappropriate treatment selection.

In spite of approval of β-lactamase inhibitors, treatment options remain limited for difficult-to-treat resistant (DTR) and multidrug resistant (MDR) bacteria, such as *Pseudomonas aeruginosa*, *Stenotrophomonas maltophilia*, carbapenem-resistant (CR) *Acinetobacter baumannii,* or CR Enterobacterales, which have become increasingly resistant to existing antimicrobial measures [1]. 

Cefiderocol (CFD) was first approved in April 2020 for CR GNB infections with limited treatment options. It is a novel siderophore cephalosporin that mimics naturally occurring siderophore molecules, which are iron-chelating molecules that are produced and released by bacterial species to facilitate iron transport for survival and growth. By exploiting this iron-transport system, CFD gains active entry into periplasmic space. Once inside, it dissociates from iron, and binds to penicillin-binding proteins, thereby inhibiting cell wall synthesis, which in the end leads to cell death. Furthermore, it has also demonstrated structural stability against hydrolysis by serine- and metallo-β-lactamases [2,3,4]. 

Several studies have demonstrated a significant association between early administration of CFD and improved clinical outcomes, supporting its role as an appropriate therapeutic option for challenging GNB infections when limited treatment options [3,5,6,7]. However, there are currently limited data regarding the use of CFD in Hospital at Home (HaH) settings [5,6], and no previous experience with this have been reported. At present, HaH programs are equipped with the infrastructure and devices required to ensure the safe administration of CFD [8,9,10], yet real-world evidence remains scarce. To our knowledge, this is the first article describing CFD use across multiple Spanish HaH units.

## 2. Results

### 2.1. Demographic Data

From January 2023 to December 2024, 27 patients were included from six centers, all from Spain. Demographics and comorbidities are depicted in Table 1, noting that the median age was 69 years (interquartile range [IQR: 53.0–80.6]), and 70.4% of patients were male. The median Charlson comorbidity index was 3 (IQR: 2.5–7), and the most common comorbidities were diabetes mellitus (37%), chronic lung disease (33.3%), chronic renal failure (25.9%), active solid cancer (22.2%), hemiplegia (18.5%), and peripheral vascular disease (14.8%). A total of 40.7% of patients were immunosuppressed.

Most patients were referred from a conventional hospital ward (74.1%), followed by a day-hospital (14.8%), primary care (7.4%), and lastly an emergency department (3.7%).

### 2.2. Microbiology

Information on infection acquisition and microbiology data is available on Table 2. As detailed, most infections were nosocomial (65.4%), with skin and soft tissue infections being the most common (37%), followed by abdominal infections (22.2%). Notably, 14.8% of patients presented with septic shock on arrival. Among all microbiological samples collected, 33.3% corresponded to swabs or exudates from skin and soft tissue lesions, 18.5% to urine cultures, and 14.8% to respiratory samples (including sputum and bronchoalveolar lavage).

**Figure 1:**
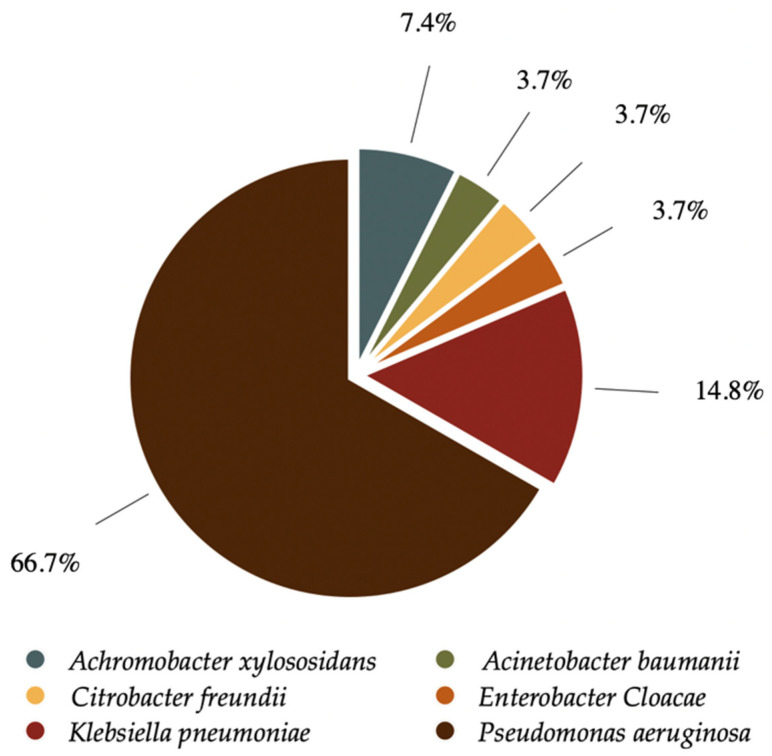
Distribution of the most frequent isolated microorganisms identified.

The distribution of microorganisms identified among the samples is detailed in Figure 1 and Table 3, with *Pseudomonas aeruginosa* (66.6%) being the most common pathogen, followed by *Klebsiella pneumoniae* (14.8%). In 14 patients, more than one pathogen, not necessarily a GNB, was identified within the same clinical sample, with the most frequent being *Enterococcus faecalis*, *Pseudomonas aeruginosa*, and methicillin-resistant *Staphylococcus aureus*.

**Figure 2:**
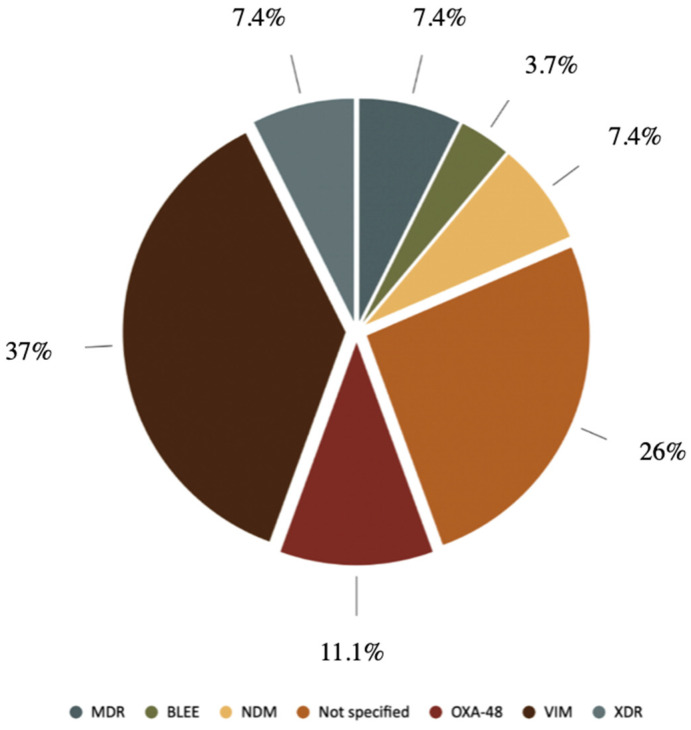
Representation of the most frequently identified resistance mechanisms.

Resistance mechanism data were available for 20 patients (Figure 2 and Table 4). Among these, 50% carried the Verona integron-encoded metallo-B-lactamase (VIM), 15% expressed oxacillinase (OXA-48) enzymes, and 10% harbored New Delhi metallo-B-lactamase (NDM). Additionally, 5% produced extended-spectrum-B-lactamases (ESBLs). Regarding resistance profiles, 10% of isolates were classified as multidrug-resistant (MDR) and another 10% as extensively drug-resistant (XDR). These resistance profiles supported the use of CFD as a targeted therapy.

### 2.3. Cefiderocol Monitoring and Use

Prior to CFD initiation, 55.5% of patients had received previous antibiotic therapy, with a mean duration of 11.8 days. CFD was administered as a first-line therapy in 63% of cases and was guided by susceptibility testing according to microbiological results in all cases. Monotherapy was the most frequent regimen; however, combined therapy was used in 40.7% of cases, most commonly with oxazolidinones and fluoroquinolones (mainly ciprofloxacin).

CFD was administered following a standardized daily dose of 6000 mg, corresponding to the approved regimen of 2 g every 8 h (q8h) according to the current guidelines. Across participating centers, CFD was delivered either as extended infusions every 8–12 h or as a continuous 24 h infusion, depending on each hospital’s local protocol. Infusions were administered with either an electronic infusion pump (52% of patients) or elastomeric device (48%). In patients with renal impairment, dosage adjustments were applied: two patients with an estimated glomerular filtration rate (eGFR) of 30–60 mL/min received 4500 mg/day (equivalent to 1.5 g q8h), and three patients with an eGFR of 15–20 mL/min received 3000 mg/day (1g q8h). Renal function and dosing adjustments were based on creatinine clearance estimated using the CKD-EPI formula. The mean treatment duration was 21.7 days, with a median of 14 days (IQR 10–28 days), whereas the mean treatment duration while in HaH was 16.37 days. Notably, three patients (11%) self-administered the entire course of CFD therapy, following appropriate training and clinical monitoring.

Regarding venous access, the most frequent type of access used was peripherally inserted central catheter or PICC (33.3%), followed by peripheral venous conventional catheter (29.6%), 20 cm midline (22.2%), central venous catheter (11.1%) and other type of access (3.7%). 

### 2.4. Safety and Outcomes

CFD treatment was well tolerated: adverse drug reactions (ADRs) occurred in two patients [7.4%, renal failure (*n* = 1) and cutaneous mycosis (*n* = 1)], leading to treatment discontinuation in one case.

The most common reason for CFD discontinuation was clinical success, which was achieved in 24 of 27 patients (88.8%; 95% CI 70.8–97.6); while adverse effects and clinical worsening each accounted for 3.7% [(*n* = 1), 95% CI 0.9–18.9], respectively.

Among the 26 patients with available data, two patients required escalation of care with hospital readmission (7.7%; 95% CI 0.9–25.1)—these episodes were all-cause—and one patient needed a <24 h emergency department visit (3.8%, 95% CI 0.1–19.6). No patient died during admission. Five patients were readmitted within 30 days post HaH discharge for all-cause reasons (19.2%; 95% CI 6.6–39.4). Infection-related recurrence at 30 days was documented in 2 out of 26 patients, corresponding to a recurrence rate of 7.7% (95% CI 0.9–25.1). At the follow-up, 8 patients had died (30.8%; 95% CI 14.3–51.8); none of the deaths were infection-related, and none occurred within the first 30 days after HaH discharge.

## 3. Discussion

In recent decades, the management of complex GNB infections has undergone a paradigm shift, with HaH and OPAT programs emerging in an increasingly prominent role in clinical practice, antimicrobial stewardship, and patient-centered care [11]. Several systematic reviews have documented the expanding implementation of HaH with home-OPAT for the treatment of multidrug-resistant GNB infections, reporting clinical outcomes comparable to those achieved with conventional inpatient management. For instance, a 2023 meta-analysis including 7539 patients receiving home-OPAT and 3857 patients treated in conventional hospitalization demonstrated no significant differences in readmission rates (OR 0.095, 95% CI 0.77–1.18) or treatment failure [12]. Moreover, multiple reviews assessing the safety and efficacy of home-OPAT as an alternative to inpatient therapy have concluded that, when combined with rigorous patient selection, structured monitoring, and standardized protocols, this approach constitutes a safe and effective strategy for the management of such infections [13]. 

Within this framework, it is relevant to examine the role of novel molecules with activity against multidrug-resistant GNB, such as CFD. CFD is administered parenterally by intravenous infusion over 3 h, following reconstitution and dilution with solutions such as 0.9% sodium chloride or 5% dextrose [14]. Several studies have demonstrated physicochemical stability, remaining stable for up to 6 h at room temperature (25 °C) and up to 24 h under refrigeration (2–8 °C, protected from light). Recent data also indicate that CFD maintains stability for up to 24 h at higher temperatures (32–37 °C), which are closer to those reached within elastomeric pumps, further supporting its potential use in HaH programs [15]. It is recommended that the infusion is initiated within the first 6 h after removal from refrigeration [10,14,16]. This administration profile aligns well with the organizational structure and workflow of HaH programs, as available devices such as refrigerated electronic or elastomeric pumps allow for accurate parenteral delivery while preserving drug stability, positioning CFD as a safe and feasible option for use in this setting. 

In line with this, HaH programs already have a well-established organizational structure, both in Spain—through the Spanish Society for Home Hospitalization (SEHAD)—and in other European countries and the United States. The official SEHAD guidelines [9] define criteria for patient selection, recommended vascular access devices, clinical and laboratory monitoring requirements, surveillance of adverse reactions, and coordination with hospital pharmacies for antimicrobial preparation and stability in different infusion systems. This established and validated infrastructure supports the feasibility and safety of integrating novel antibiotics, such as CFD, into HaH programs [8,9].

Although the existing organizational structure facilitates the administration of CFD within these programs, scientific evidence on its use in this setting remains limited globally and is currently lacking in Spain. In February 2024, Schellong et al. reported a case of osteomyelitis caused by *Pseudomonas aeruginosa* producing NDM, initially treated with ampicillin/sulbactam for 28 days and subsequently with CFD for 37 days in a conventional hospital setting. The patient was then transferred to HaH to complete an additional 63 days of antimicrobial therapy via PICC, with no complications and favorable clinical outcomes [4]. A year later, in May 2025, Babich et al. described another case demonstrating the technical feasibility of continuous CFD infusion using an elastomeric pump. The patient, who had otomastoiditis caused by *Klebsiella pneumoniae* producing NDM, achieved adequate pharmacokinetic/pharmacodynamic (PK/PD) targets, allowing therapeutic drug monitoring at home in a patient with good clinical results and no reported complications [16].

To date, only a few cases of continuous CFD infusion have been reported, most of them in patients receiving renal replacement therapy. In our study, nearly half of the cohort was treated with continuous infusion, providing relevant safety data that support this administration mode as a potentially practical and effective strategy in selected scenarios. Recent in silico pharmacokinetic modeling by Goutelle et al. compared short, prolonged, and continuous infusion regimens, suggesting that continuous infusion may optimize the percentage of time that the free drug concentration remains above the minimum inhibitory concentration (MIC) and maintain target exposure, particularly in critically ill patients [17].

Published evidence with CFD in HaH settings have been limited to isolated case reports and theoretical PK/PD modeling, with no structured cohorts available. While previous reports have demonstrated technical feasibility, these descriptions involved single patients and provide only anecdotal insights. Similarly, in silico PK/PD simulations have suggested that continuous infusion may enhance target attainment, but these studies lacked real-world clinical validation. Our multicenter Spanish cohort therefore represents the first systematic evaluation of CFD in an HaH context, offering real-world evidence that complements and expands upon these preliminary observations.

By providing multicenter safety data, feasibility outcomes, and effectiveness measures in a heterogeneous population of patients with multidrug-resistant GNB infections, our results move beyond theoretical and establish a practical framework for implementing CFD within home-based care models. This contribution helps bridge the gap between pharmacological predictions and actual program-level implementation, positioning CFD as a valid therapeutic option for complex infections managed outside a hospital setting. 

In our cohort, nearly half of the patients received treatment through electronic infusion devices, and 11% even self-administered therapy throughout the course of treatment, which likely enhanced adherence and optimized healthcare resource utilization. This approach facilitates patient management in the home environment, improving comfort and quality of life while avoiding prolonged hospitalization. 

The results demonstrate that CFD represents a valid and safe therapeutic alternative for patients with difficult-to-treat GNB infections, achieving high clinical success rates (88.8%) and low incidence of adverse events (7.4%). In addition, the low 30-day recurrence rate (7.7%) further supports its effectiveness. During HaH management, escalation of care was required in 7.7% of patients, a figure slightly higher than the readmission rate typically reported for HaH programs. However, this finding is consistent with the greater clinical complexity of the population included in this study, which primarily comprised patients with multidrug-resistant GNB infections.

Rather than representing an isolated experience, our findings advocate for the integration of an innovative antimicrobial agent into an established and safe model of care, paving the way for new strategies in home-based management of complex infections.

Finally, several limitations should be acknowledged when interpreting these findings. Although this was a multicenter study, the sample size was relatively small, which limits the statistical power and the generalizability of the results. Moreover, the descriptive and retrospective design precludes the establishment of causal relationships or direct comparisons of efficacy with other therapeutic options, and certain data were not consistently available across all centers. Therefore, these findings should be interpreted with caution and confirmed through larger, prospective, multicenter studies.

## 4. Materials and Methods

This was a retrospective, observational study conducted in 6 Spanish centers from January 2023 to December 2024. Spanish centers were recruited through the Spanish Hospital at Home Society (SEHAD) and the Spanish Internal Medicine Society (SEMI) HaH workgroup. Inclusion criteria were adult patients with GNB-related infections treated with CFD in HaH for at least 4 doses. The exclusion criteria were patients not fitting the inclusion criteria and those who refused admission to an HaH program.

### 4.1. Outcomes and Definitions

The primary objective of this study was to evaluate the feasibility and efficacy of CFD use in HaH program, assessed through the practicality of different modes of drug administration, types of venous access used, clinical outcomes, recurrence rates, and treatment discontinuation. The secondary objective was to assess its safety, including the incidence and nature of adverse drug reactions (ADR), the need for escalation of care, and attributable mortality during follow-up.

ADR was defined as any unfavorable clinical event temporally related to CFD administration and not reasonably explained by the patient’s underlying condition or other concomitant therapies. Treatment discontinuation was defined as the premature interruption of CFD due to either clinical success, ADR, microbiological readjustment, or clinical worsening. Clinical success was defined as resolution of the infection leading to CFD initiation. Microbiological success was assessed based on the absence of persistence of isolates in follow-up control cultures. Escalation of care refers to the need for transfer of medical attention to a hospital setting while in HaH, both for hospital readmission or <24 h emergency department visit. Readmission at 30 days was defined as all-cause hospital admission within 30 days after discharge from the HaH program. Recurrence was defined as the reappearance of the initial infection, including the same microorganism and site, within the first 30 days after treatment completion. Finally, attributable mortality was defined as death directly related to the underlying infectious process.

### 4.2. Microbiological Methods

Microbiological identification and susceptibility testing were performed according to accredited local laboratory procedures, typically using MALDI-TOF or automated phenotypic systems. Antimicrobial susceptibility interpretation, including CFD, followed EUCAST breakpoints (version in use at each site) [18], and the diagnostic approach to MDR/XDR GNB adhered to international standards, including IDSA guidance [19]. Minimum inhibitory concentration (MIC) values for CFD were not uniformly available across participating centers and were therefore not included.

Multidrug-resistant (MDR) organisms were defined according to international consensus criteria as isolates exhibiting non-susceptibility to at least one agent in three or more antimicrobial categories. Extensively drug-resisistant (XDR) organisms were defined as isolates remaining susceptible to no more than one or two antimicrobial categories, with resistance to all others.

### 4.3. Statistical Analysis

Patient data were anonymized, coded, and subsequently included in a shared database. Descriptive statistics are expressed to summarize the cohort’s characteristics. Categorical variables are expressed as frequencies and percentages, whereas continuous variables are expressed as mean +/− standard deviation or median with interquartile range. No statistical correlation analysis was performed, as the study design was primarily descriptive and the sample size limited the feasibility of inferential statistics. Descriptive analyses were performed using Microsoft Excel (Microsoft Corporation, Redmond, WA, USA). 

## 5. Conclusions

This multicenter descriptive study highlights the use of CFD within HaH programs for the management of difficult-to-treat GNB infections, demonstrating it to be a feasible and safe option. The study included 27 patients from six Spanish centers, to whom treatment was delivered following a standardized regimen—most frequently via PICC and electronic pump infusion devices—and, in selected cases, even self-administered, underscoring the adaptability of this model.

Our findings, although limited by our observational design and sample size, contribute to a field in which real-world evidence remains scarce, supporting the integration of this novel molecule into consolidated HaH programs and encouraging further multicenter research to confirm these findings.

## Figures and Tables

**Table 1:** Patient demographic data and comorbidities.

	Demographics and Comorbidities (*n* = 27)	Number, %
Patient characteristics	Age, years; median (IQR)	69 (IQR: 53–80.6)
	Sex	
	Female	8, 29.6%
	Male	19, 70.4%
	Charlson comorbidity index; median (IQR)	3 (IQR: 2.5–7)
Comorbidities	Immunosuppression	11, 40.7%
	Diabetes mellitus	10, 37%
	Chronic lung disease	9, 33.3%
	Chronic renal failure	7, 25.9%
	Active cancer	6, 22.2%
	Hemiplegia	5, 18.5%
	Peripheral vascular disease	4, 14.8%
Referral department	Hospital ward	20, 74.4%
	Day-hospital	4, 14.8%
	Primary care	2, 7.4%
	Emergency department	1, 3.7%

**Table 2:** Infection presentation.

	Infection Presentation	Number, %
Infection acquisition (*n* = 26)	Nosocomial	17, 65.4%
	Community-acquired	9, 34.6%
Type of infection (*n* = 27)	Skin and soft tissue infections	10, 37%
	Abdominal infections	6, 22.2%
	Urinary tract infections	5, 18.5%
	Respiratory tract infections	4, 14.8%
	Osteoarticular	2, 7.4%
Microbiological samples (*n* = 27)	Swabs/Exudates skin and soft tissue lesions	9, 33.3%
	Urine culture	5, 18.5%
	Respiratory samples	4, 14.8%
	Blood culture	3, 11.1%
	Peritoneal fluid samples	2, 7.4%

**Table 3:** Distribution of Gram-negative isolates identified in the study.

Microorganism	Isolate	Number
GNB	*Achromobacter xylosoxidans*	2
	*Acinetobacter baumanni*	1
	*Citrobacter Freundii*	1
	*Enterobacter cloacae*	1
	*Klebsiella pneumoniae*	4
	*Pseudomonas aeruginosa*	18

**Table 4:** Distribution of resistance mechanisms and phenotypic resistance profiles identified among the isolates.

Resistance Mechanism	Number
VIM	10
OXA-48	3
NDM	2
BLEE	2
KPC	1
Phenotypic resistance group	Number
MDR	2
XDR	2

## Data Availability

The data presented in this study are available on request from the corresponding author. The data are not publicly available due to privacy and ethical restrictions.
